# Ag-Coated Cellulose Fibers as Surface-Enhanced Raman Scattering Substrates for Adsorptive Detection of Malachite Green

**DOI:** 10.3390/ma11071197

**Published:** 2018-07-12

**Authors:** Yudong Lu, Changji Wu, Yang Wu, Ruiyun You, Gang Lin, Youqiang Chen, Shangyuan Feng

**Affiliations:** 1 College of Chemistry and Materials Science, Fujian Key Laboratory of Polymer Materials, Fujian Normal University, Fuzhou 350117, China; 18060489009@163.com (C.W.); 13506970317@163.com (Y.W.); youruiyun@fjnu.edu.cn (R.Y.); 2 Center of Engineering Technology Research for Microalgae Germplasm Improvement of Fujian, Southern Institute of Oceanography, Fujian Normal University, Fuzhou 350117, China; yqchen@fjnu.edu.cn; 3 Key Laboratory of Optoelectronic Science and Technology for Medicine of Ministry of Education, Fujian Provincial Key Laboratory of Photonics Technology, Fujian Normal University, Fuzhou 350117, China; syfeng@fjnu.edu.cn

**Keywords:** surface-enhanced Raman scattering, silver, cellulose, Malachite green

## Abstract

Surface-enhanced Raman scattering (**S**ERS) is a sensitive technique for the detection of low concentration analytes. In this study, we used cellulose fibers (CF) as the templates for the loading of silver nanoparticles (Ag NPs), and the obtained CF-Ag was applied in the detection of R6G and Malachite Green (MG) by surface-enhanced Raman scattering. The adsorption technique was employed in the sample preparation, and the optimal detecting status was identified in the dynamic range (sample status ranging from wet to dry) for different concentration of analytes. In comparison to Ag NPs, CF-Ag showed enhanced performance for adsorptive detection of Malachite Green, and the limit of detection was 5 × 10^−12^ M.

## 1. Introduction

Malachite green (MG), with a triphenylmethane core, is commonly used as a dye in the textile, leather, and papermaking industries [1]; it also is used as a water-soluble bactericide in aquacultures. However, MG is banned in many countries because it is easily absorbed into the human body and metabolizes into highly toxic residues that are carcinogenic and teratogenic [2]. Although MG can be employed in diluted form as a local antiseptic, its use as a drug is prohibited in the USA, Canada, and several countries in Europe [3]. Additionally, the China National Standard stipulates that the MG content in water breeding products must be below 1 μg/kg. Moreover, because of its adverse effects on water breeding products, several methods have been developed to detect MG. These include techniques such as adsorptive stripping voltammetry [4], high-performance liquid chromatography (HPLC) [5], immunoassays [6], mini-column extraction [7], molecular imprinting [8,9], and surface-enhanced Raman spectroscopy (SERS) [10]. In a study by Sun [11], a PMMA (polymethyl methacrylate)/Ag/graphene/Ag/graphene substrate was used to detect MG with a sensitivity of 10^−7^ M. Moreover, Kumar et al. [10] prepared a Ag-coated PDMS SERS substrate using taro leaf as a template, achieving a very low MG detection limit of 10^−11^ M, which could be attributed to the high local field produced by the Ag granular film and nanovoids on the PDMS surface. However, in this case, the characteristic peak was too weak to be detected in the spectrum less than 10^−8^ M MG.

SERS [12,13,14] is a highly sensitive, nondestructive, specific, and rapid analytical method, which provides abundant information about organic compounds [15]. The technique depends on molecular adsorption on the surface of a metal-gel nanostructure, such as Ag nanoparticles (NPs) [16], Au NPs [17,18], or other nanoscale species like arrays with Ag nanocubes [18]. In addition, eco-friendly and low-cost SERS substrates have drawn considerable attention from researchers. These include biomimetic materials [10,19], paper-based materials [20,21], fibers [20,21], and egg shell membranes [22]. For example, in a study by Huang, Ag was sprayed onto a taro leaf [23] as an SERS substrate; it demonstrated that this substrate has a detection limit as low as 10^−8^ M for R6G (Rhodamine 6G) dyes. Moreover, Yu [24] and Xiao [25] printed Ag NPs onto filter, chromatography, and weighing paper as ink for SERS detection. 

Because of its porous structure and surface functional groups, cellulose has been frequently used as a carrier for the synthesis of various kinds of nanoparticles in situ. For example, Pang et al. [26] synthesized one-dimensional nanocrystals with diverse morphologies using cellulose as a nanoreactor. Moreover, other reports have presented the use of bamboo hemicellulose [27] and hydroxypropyl cellulose [28] as templates to synthesize Ag NPs, demonstrating that Ag^+^ readily combines with O^−^ in the R-OH or ROOH groups of cellulose. Hence, in consideration of the cost and eco-friendliness, many SERS substrates have been synthesized using bacterial cellulose (BC), vegetable cellulose (VC) [29], or nanocellulose [30]. Marques et al. [29] compared two substrates, which used VC and BC as templates. Results indicated that the BC-Ag material showed a much lower detection limit that VC-Ag because some of the dissolved target molecules were trapped in the thin fibers of the material. Additionally, Liou et al. [30] prepared cellulose fibers coated with Ag, demonstrating their use for the detection of 4-aminothiophenol (pATP) and thiabendazole (TBZ) at a detection limit of 1 ppm, i.e., 10^−5^ M level. Additional, the paper-based SERS substrate [31] was prepared and applied for detecting HPV, but its process of preparation was complex.

In this work, we synthesized CF-Ag via Ag NPs in situ reduction with the use of cellulose fibers as templates. After heat treatment of the mixture, which contained AgNO_3_ and CF, Ag seeds were formed on the surface of CF. The obtained CF-Ag was used to detect R6G and Malachite Green (MG) by surface-enhanced Raman scattering, and it exhibited much higher detection sensitivity than Ag nanoparticles without cellulose fibers.

### 2.1. Reagents and Apparatuses

CFs solution (2–6%) was provided by Chemkey Advanced Material Technology Co. Ltd. (Shanghai, China). Silver nitrate (purity ≥99.8%), hydroxylamine hydrochloride (purity ≥99.8%), sodium hydroxide, and MG were obtained from Sinopharm Group (Shanghai, China), Rhodamine 6G was obtained from MACKLIN (Shanghai, China). All solutions were prepared by using Millipore water (Guozhiyuan Y1/2-10UV, Kertone, Changsha, China) as the solvent. Aluminium metal sheet (AR) was obtained from Fuchen chemical reagents Co., Ltd. (Tianjin, China). For analysis, we used the JEM 2100 transmission electron microscope (JEOL Ltd., Tokyo, Japan), the UV1902 UV-Vis spectrometer (Lengguang Tech., Shanghai, China) and Regulus 8100 scanning electron microscope (HITACHI, Tokyo, Japan).

### 2.2. Preparation of CF-Ag NPs

First, 15 mL of AgNO_3_ solution with different concentration (6 mM, 12 mM, 15 mM, and 18 mM) was slowly added to 15 mL of 4% CF solution, and the obtained mixture was stirred slowly for 30 min to ensure that Ag^+^ was well adsorbed on the surface of CF. Then, the suspension was heated to boiling point and was left at that temperature until its color turned orange. It was subsequently left to cool down naturally to room temperature. After that, a mixture of 4.5 mL 0.1 M NaOH and 5 mL 0.06 M HO-NH2·HCl was poured into the system under vigorous stirring at room temperature (15 °C). The gentle stirring was continued for 20 min and the formed suspension was denoted as CF-Ag NPs. For control experiment, Ag NPs was prepared by the same procedure, without adding CF.

### 2.3. Sample Preparation

2 µL CF-Ag NPs or Ag NPs was dispersed into 1 mL of MG solutions with different concentrations (10^−12^~10^−8^ M). After the solutions were fully mixed, the mixture was centrifuged and the supernatant liquid was removed. The sediment was redispersed in water to form suspension (7 µL). 

### 2.4. Measurement

SERS spectra were acquired by a Renishaw confocal (Wotton-under-Edge, UK) Raman instrument equipped with a 785 nm laser and 20× objective. The accumulation time for each measurement was 10 s.

### 2.5. Date Analyst

A Vancouver Raman algorithm [32], based on a fifth-order polynomial fitting method, was used to remove fluorescence background and noise signals for all the raw SERS spectra. The Origin 8. (Originlab, Hampton, MA, USA) was used for data analysis. 

## 3. Results and Discussion

**Figure 1:**
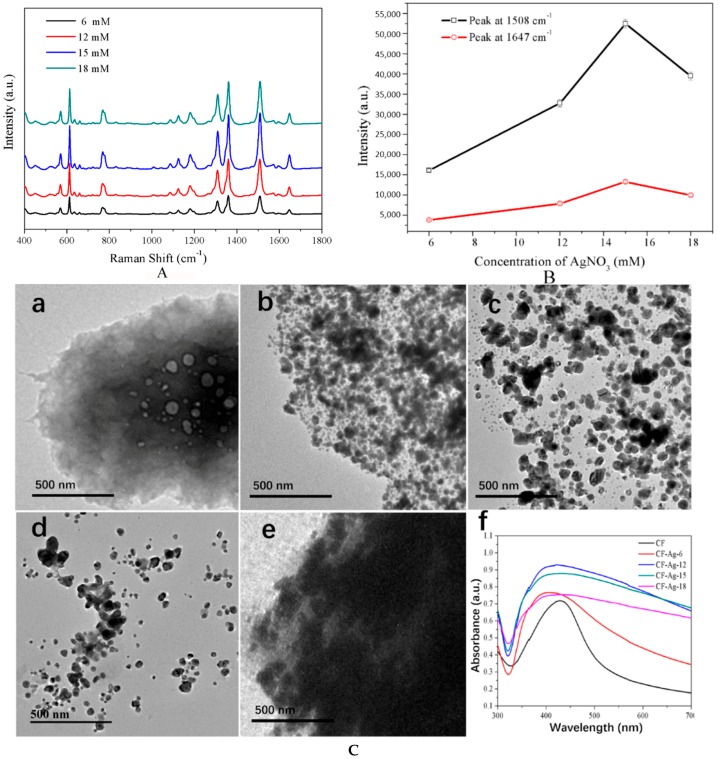

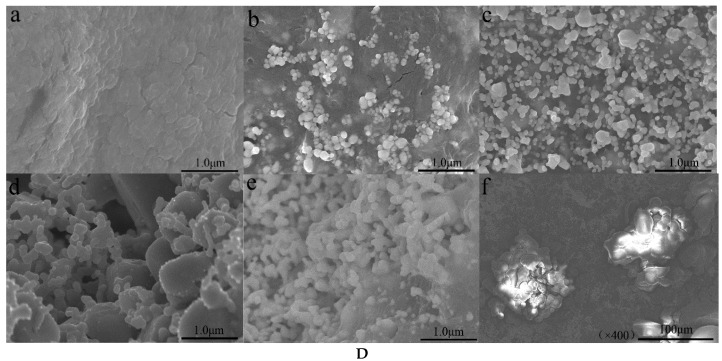
(**A**) SERS spectra of 10^−4^ M R6G detected with CF-Ag prepared with 6, 12, 15, and 18 mM AgNO_3,_ and CF (spectrum above is the average of five measurements); (**B**) Peaks at 1508 and 1647 cm^−1^ in the SERS spectra shown in A; (**C**) (a–e) TEM images of CF, CF-Ag-6, CF-Ag-12, CF-Ag-15, and CF-Ag-18 (“6, 12, 15, 18” indicates that 6 mM, 12 mM, 15 mM, and 18 mM AgNO_3_ were used, respectively) and (f) UV adsorption spectra of CF-0 (Black), CF-Ag-6 (Bright red), CF-Ag-12 (Blue), CF-Ag-15 (Green), and CF-Ag-18 (Plum Purple). (**D**)SEM of images of (a) CF, (b) CF-Ag-6, (c) CF-Ag-12, (d) CF-Ag-15, (e,f)and CF-Ag-18. (×30,000 with (a–e), and ×400 with (f)).

In an effort to identify the optimal AgNO_3_ content in the material for effective dye detection, 6, 12, 15, and 18 mM AgNO_3_ were used to prepare CF-Ag, which were employed as SERS substrates in the detection of 10^−4^ M R6G (Figure 1A). All spectra clearly exhibited peaks due to R6G at 770 cm^−1^, 1182 cm^−1^, 1310 cm^−1^, 1362 cm^−1^, 1508 cm^−1^, 1575 cm^−1^, and 1647 cm^−1^ [33]. The intensity strength of R6G increased with an increase in AgNO_3_ concentration until it reached 18 mM, with CF-Ag-15 obtaining the strongest enhancement of the R6G spectra. Figure 1B provides additional details on the variation of the intensities of the peaks at 1508 and 1647 cm^−1^. Clearly, with the increase in AgNO_3_ concentration, there was first an increase and then a decrease in intensity, with CF-Ag-15 as the most intense peak (Figure 1A). Moreover, as seen in the TEM images in Figure 1C, with the increase in AgNO_3_ concentration, the Ag NPs increased in size. However, with excess Ag^+^ present (Figure 1C(e)), the small Ag seeds may reunite to form larger ones after the addition of the reducing agent. This may lead to a larger density of Ag NPs, which would make the gap between the Ag NPs too narrow to sufficiently adsorb molecules. Ultraviolet adsorption spectra showed that the peak due to CF was narrower than CF-Ag NPs as a result of the aggregation of Ag NPs upon growth as well as the location of the UV peak on the cellulose; concurrently, the width of the peak depended on the Ag NPs (Figure 1 C(f)). Therefore, CF-Ag-15 offers the optimal Ag NPs nanoscale and density for enhanced SERS. Additionally, the SEM image provides a better depiction of the results. The density of Ag NPs increased as the AgNO_3_ increased. In Figure 1 D(c,d), the number of Ag NPs increases and the particle size becomes bigger. When the concentration of AgNO_3_ continues to increase, the exceeding Ag+ ensures that the Ag NPs continue to grow, increasing in both density and size. Further, the enhancement factor of CF-Ag-15 was calculated to be 1.89 × 10^8^ at 1508 cm^−1^ and 2.15 × 10^8^ at 1362 cm^−1^ respectively.

The precise calculation process is as follows [34]:

(1) $$\mathrm{EF}=\frac{I_{\mathrm{SERS}}\;\times N_{\mathrm{bulk}}\;}{I_{\mathrm{RS}}\;\times N_{\mathrm{SERS}}}\;$$

where I_SERS_ is the SERS intensity of the analyte (in this case, R6G) mixed with CF-Ag-15. I_Raman_ is the normal Raman intensity of the R6G measured over an aluminum sheet. N_SERS_ is the number of molecules probed in SERS. N_bulk_ is the powder of the analyte sample.

N_SERS_ = CVN_A_S_scan_/S_sub_ (2)

N_bulk_ = MρhN_A_A_Raman_ (3)

C is the molar concentration for analyte solution, V is the volume of the droplet = 5 μL in SERS, S_scan_ is the area of Raman scanning, S_scan_ = 6.25 μm^2^ and S_sub_ is the area of the substrate; S_sub_ = 4.9 mm^2^ (d = 2.5 mm, nearly circular). A_Raman_ is the laser spot diameter, which was calculated for 785 nm using the formula, with the laser spot diameter = (1.22λ/N_A_), N_A_ = 0.4. A_Raman_ = 2.4 μm and h is the confocal depth with 20× objective, h = (2λ/NA^2^) = 9.8 μm. I_SERS_ = 76780, I_RS_ = 322.6 at 1360 cm^−1^, C = 2.5 × 10^−5^ M,

So N_SERS_ = (2.5 × 10^−5^ × (5 × 10^−6^) × 6.02 × 10^23^ × 6.25 × 10^−12^)/4.9 × 10^−7^ = 9.6 × 10^7^;

N_bulk_ = 479 × 1.25 × (9.8 × 10^−6^) × (6.02 × 10^23^) × (6.25 × 10^−12^) = 8.48 × 10^14^ (4)

(5) $$EF=\frac{76780\times 8.48}{322.6\times 9{.6\times 10}^{7}}=2.11\times 10^{8}\;$$

Similarly, $EF=1{.85\times 10}^{8}$ at 1501 cm^−1^.

**Figure 2:**
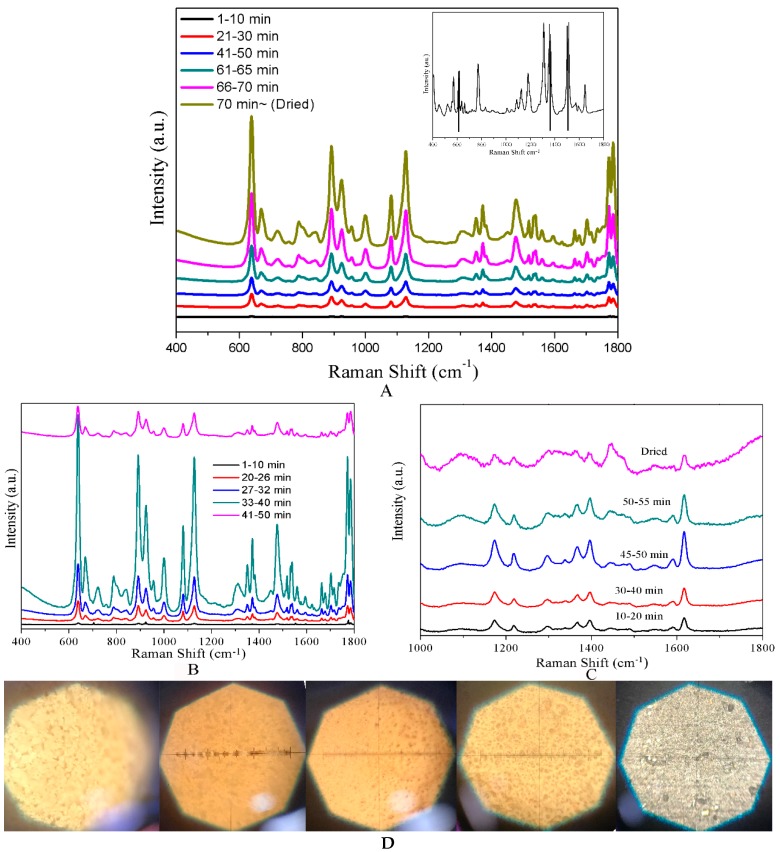
(**A**) SERS spectra for dynamic detection of 5 × 10^−5^ M MG. (**B**) SERS spectra of 5 × 10^−6^ M MG detected with CF-Ag prepared using 15 mM AgNO_3_ from wet to dry. (**C**) SERS spectra of 10^−7^ M MG detected with CF-Ag prepared using 15 mM AgNO_3_ from wet to dry (spectra above were averaged from five measurements). (**D**) Photographs of CF-Ag recorded via a microscope from wet to dry.

When the optimum CF-Ag NPs were obtained, it was used for MG research. In Figure 2A, all the spectra were obtained at a laser power of 0.66 mW, with 10^−5^ M R6G over 1–70 min. Prominent peaks were found at 427, 445 (out-of-plane vibrations of phenyl-C-phenyl), 1172 (in-plane vibrations of ring C-H), 1395 (*N*-phenyl stretching), and 1618 cm^−1^ (ring C-C stretching) [35]. This led to stronger peaks in the spectrum, which were recorded with a 0.09 mW laser beam, (1–70 min), with an intensity occasionally exceeding the detecting range (inset in Figure 2A). The intensity of the peaks in the spectra of 70–75 min was recorded with a 0.04 mW laser beam and normalized to be 0.09 mW. Therefore, optimal spectra were obtained in a dry status of the sample. As noted previously, both the Ag NPs and CFs have a layered 3D structure. When the solvent (water) was evaporated, the distance between the CFs began to decrease; this narrowed the gap between the CF-Ag NPs [36,37], which was expected to produce hot spots. In the course of research, an unusual trend in the SERS response was seen at lower R6G concentrations; a very weak spectrum was obtained for 10^−7^ M R6G at dry than at a specific wet status. Accordingly, the influence of sample drying time was also explored over 10^−6^ M and 10^−7^ M MG. As indicated by the results of the measurements shown in Figure 2B,C, the intensity of the spectral peaks first increased and then decreased in the process from wet to dry. A portion of the intense increase was the same as the result with 10^−5^ M MG. When the sample was wet, the CF-Ag was porous and dispersed in water; only a few analyte molecules were exposed to the laser. As the drying time was extended, the evaporated water increased the distance between CF-Ag NPs in each layer, which may lead to the generation of additional hot spots. Meanwhile, the eliminated water also brought the CF closer together, resulting in the exposure of additional CF-Ag NPs as well as analyte molecules to the laser, thus increasing the spectral intensity. Notably, the sample that was nearly dry provided the most intense spectra (33–40 min in 10^−6^ M and 45–50 min in 10^−7^ M, with the time dependent on room temperature). However, upon complete drying, the gradually decreased solvent took many analytes into the aperture of the cellulose and was covered by CF, which contributed to a weaker signal. Photographs of the CF-Ag recorded via a microscope from wet to dry are shown in Figure 2C. Notably, this phenomenon presented during the detection of 10^−^^6^ M and 10^−7^. MG. But, owing to the large number of molecules saturated via adsorption on the surface of CF-Ag NPs, the number of molecules brought into the aperture of the cellulose had no significant influence.

**Figure 3:**
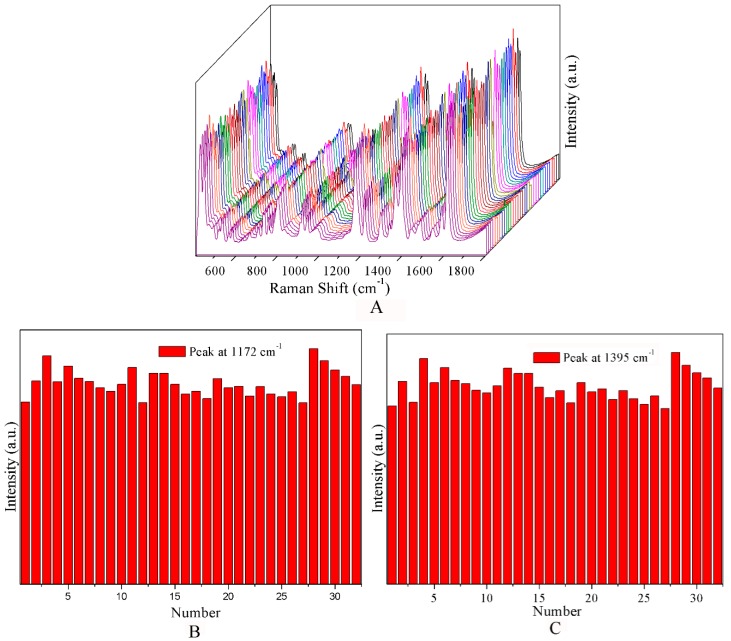
(**A**) SERS spectra of different concentrations of MG. (**B**,**C**) Graphs of the intensity of the peaks at 1172 and 1395 cm^−1^ from 32 SERS spectra of the dried sample of (**B**).

To estimate the homogeneity of CF-Ag NPs, 32 spectra randomly selected were obtained and were recorded for one sample (5 × 10^−4^ M MG) in the same condition of dry (Figure 3A). Additional details are shown in Figure 3C,D, which show the intensity of the characteristic Raman bands at 1172 cm^−1^ and1395 cm^−1^. The results indicated good homogeneity with RSD = 9.8% for the band at 1172 cm^−1^ and RSD = 10.2% for the band at 1395 cm^−1^ (Figure 3B,C).

**Figure 4:**
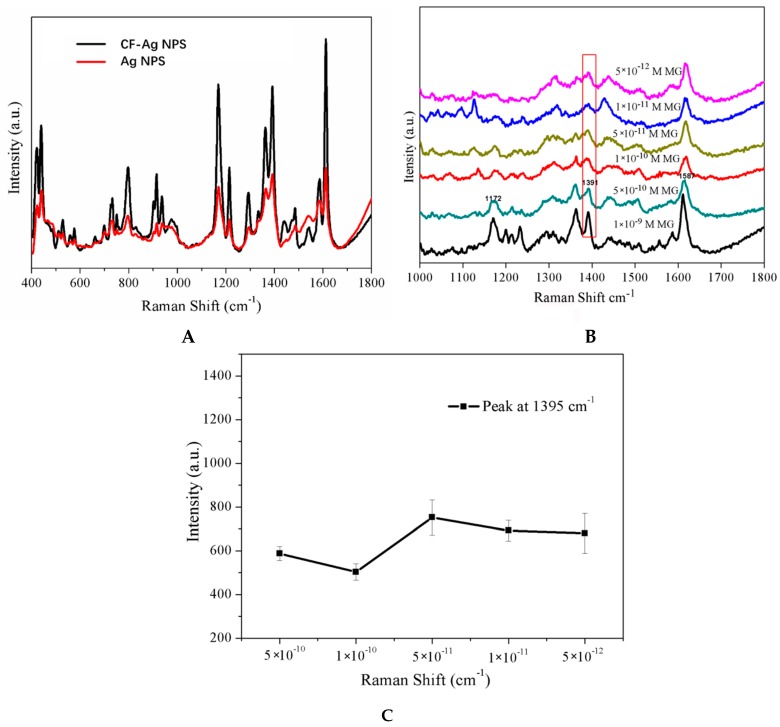
(**A**) SERS spectra of 10^−8^ M MG detected with two sample preparation methods. (**B**) SERS spectra of MG exhibiting a LOD of 10^−12^ M with absorption-method. (**C**) Peak at 1391 cm^−1^ in the SERS spectra shown in B.

According to Leng [38], MG can be adsorbed from oxygen-containing functional groups of compounds or on the surfaces of different materials. Because CF is polyporous and has many –OH functional groups and Ag NPs, it could easily adsorb analytes. As expected, the Raman signal intensity obtained from CF-Ag is much stronger than that of Ag without CF (Figure 4A). In Figure 4B, the limit of detection (LOD) for CF-Ag was 5 × 10^−12^ M MG. In addition, the characteristic band at 1391 cm^−1^ was observed (Figure 4C), implying that the intensity strength was barely changed with the change in MG concentration, possibly due to CF-Ag’s adsorption capacity. In contrast, the CF-Ag adsorption function could lead to a lower LOD, thereby exhibiting high practical utility.

## 4. Conclusions

In this study, we prepared Ag-coated cellulose fibers for SERS detection. Two types of dynamic range detection results (samples states ranging from wet to dry) were explored: the optimal detection time was obtained when the sample was completely dry for high concentration and nearly dry for low concentration. Moreover, the adsorption efficiency of the CF-Ag NPs was demonstrated for MG, with the adsorption method for sample preparation giving a lower LOD of 1 pM MG. Our study makes a significant contribution to the literature because we could detect the analyte in a lower concentration through an adsorption approach which exhibits high practical utility.
